# Hydrogel Cross-Linking via Thiol-Reactive Pyridazinediones

**DOI:** 10.1021/acs.biomac.3c00290

**Published:** 2023-10-04

**Authors:** Calise Bahou, Richard J. Spears, Angela M. Ramírez Rosales, Léa N. C. Rochet, Lydia J. Barber, Ksenia S. Stankevich, Juliana F. Miranda, Tobias C. Butcher, Adam M. Kerrigan, Vlado K. Lazarov, William Grey, Vijay Chudasama, Christopher D. Spicer

**Affiliations:** † Department of Chemistry, University College London, 20 Gordon Street, London WC1H 0AJ, U.K.; ‡ Department of Chemistry, University of York, Heslington YO10 5DD, U.K.; § York Biomedical Research Institute, University of York, Heslington YO10 5DD, U.K.; ∥ The York JEOL Nanocentre, University of York, Heslington YO10 5BR, U.K.; ⊥ School of Physics, Engineering, and Technology, University of York, Heslington YO10 5DD, U.K.

## Abstract

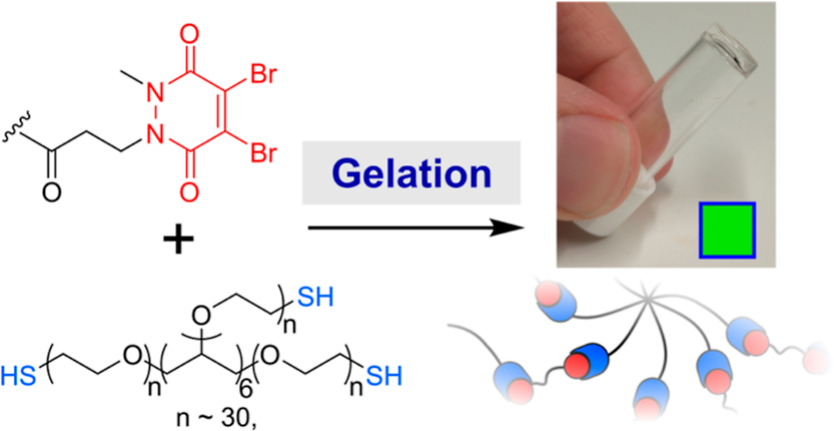

Thiol-reactive Michael acceptors are commonly used for the formation of chemically cross-linked hydrogels. In this paper, we address the drawbacks of many Michael acceptors by introducing pyridazinediones as new cross-linking agents. Through the use of pyridazinediones and their mono- or dibrominated analogues, we show that the mechanical strength, swelling ratio, and rate of gelation can all be controlled in a pH-sensitive manner. Moreover, we demonstrate that the degradation of pyridazinedione-gels can be induced by the addition of thiols, thus providing a route to responsive or dynamic gels, and that monobromo-pyridazinedione gels are able to support the proliferation of human cells. We anticipate that our results will provide a valuable and complementary addition to the existing toolkit of cross-linking agents, allowing researchers to tune and rationally design the properties of biomedical hydrogels.

## Introduction

α,β-Unsaturated carbonyl Michael acceptors are widely used in biological and materials chemistry due to the specificity and speed with which they can react with thiol-based nucleophiles.^1,2^ The use of (meth)acrylates and maleimides has been particularly widespread due to their ease of access and rapid rates of thiol-conjugation, respectively.^3^ However, each of these classes of reagents has significant drawbacks, which may hinder their use in certain applications: the rate of reaction of (meth)acrylates with thiols is typically slow,^4^ while for maleimides, their sensitivity to hydrolysis and retro-Michael instability can be limiting.^5^ As a result, researchers have focused on the development of alternative Michael acceptors in recent years, such as “next-generation” maleimides^6^ or acyclic activated alkenes.^7^

As part of this process, we have developed pyridazinediones (PDs) as attractive reagents for selective thiol conjugation.^8–11^ In the context of site-specific protein modification, we have shown that these reagents react efficiently with thiols and do not suffer from issues associated with hydrolysis at pH 6–8.^12^ Nonbromo (DiH) PDs undergo dynamic, reversible Michael addition,^13^ while for mono- and dibromo PDs, the reverse retro-Michael addition is mechanistically unfeasible, with elimination of the bromide leading to a stable alkenyl thioether product.^12^ Thiol-substituted conjugates of monoBr- and DiBr-PDs retain the ability to undergo thiol-induced cleavage, but only when exposed to a vast excess of additional thiol at basic pH, while DiBr-PDs also have the ability to undergo bis-thiol conjugation.^14^ When combined with the differing rates of reaction with thiols, the array of DiH-, monoBr-, and DiBr-PDs therefore collectively provides an array of tunable characteristics that make them well suited to applications outside of bioconjugation.

**Figure 1:**
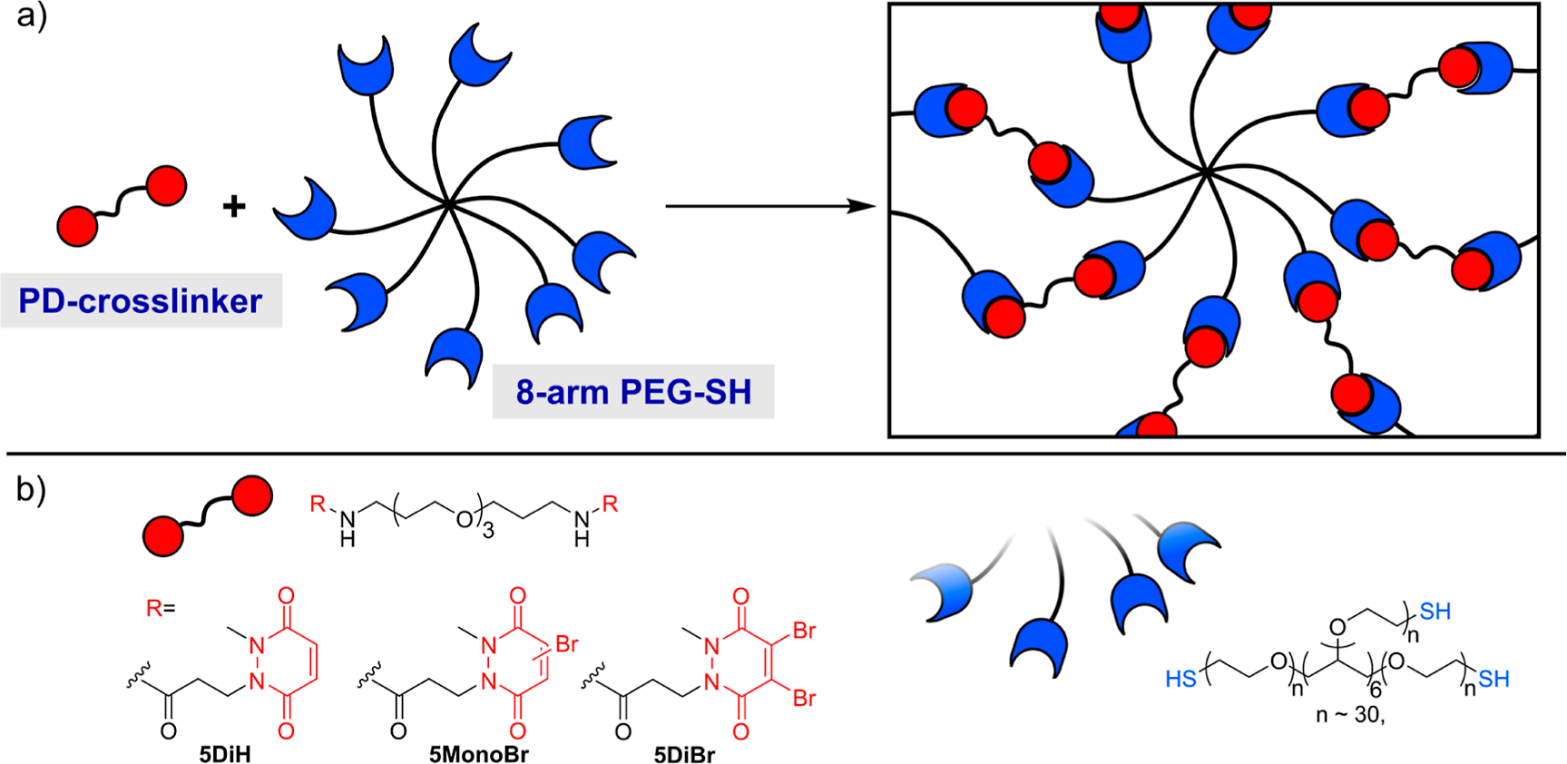
(a) Schematic overview of hydrogel formation between an 8-arm star PEG-thiol macromer (blue) and a bis-pyridazinedione (PD, red) cross-linker; (b) chemical structures of the cross-linkers and macromer used in this work.

In this paper, we realize this ambition by showing that PDs can serve as reactive handles for the construction of chemically cross-linked hydrogels (Figure 1). Although previous work in this area has focused on the use of alternative Michael acceptors such as (meth)acrylates, maleimides, and less commonly vinyl sulfones to achieve gelation, each of these reagents come with drawbacks.^3^ PDs, with their tunable and versatile reactivity, therefore represent a valuable addition to the toolkit of reagents amenable to hydrogel formulation.

## Experimental Section

### Materials

All chemicals were purchased from Merck and used as received, other than 8-arm-PEG-thiol (JenKem, 10 kDa). Details of the cross-linker syntheses and characterizations are provided in the Supporting Information.

### Gel Formation

#### General Procedure

A stock solution of the specified pyridazinedione cross-linker (400 mM) was prepared in DMSO. An aliquot of this stock (5 μL, 2 μmol, 4 μmol reactive PD) was diluted with sodium phosphate buffer (45 μL, 50 mM, pH 7.4), and the mixture added immediately to a solution of 8-arm-PEG-thiol (JenKem, 10 kDa, 5 mg, 0.5 μmol, 4 μmol reactive thiols) in sodium phosphate buffer (50 μL, 50 mM, pH 7.4). The mixture was pipetted vigorously for 2 s to mix thoroughly and then left to stand and gel at room temperature for the specified time.

#### Gelation at Different pHs

Run as described above in phosphate buffer (50 mM) at either pH 6, 7.4, or 8 in 2 mL glass vials. After 30 min, the vials were inverted and pictures taken. No gelation was observed at pH 6 at this time point. Partial gelation of **5DiH** was observed at pH 7.4, with all other mixtures being fully gelled. The inversion process was repeated after 24 h. At this time point, all mixtures had fully gelled.

### Rheology

#### Amplitude Sweep

After being left to form for 24 h, gels were transferred to the rheometry plate and the measurement gap set to 1 mm. Measurements were performed at 25 °C with a solvent trap that was used to maintain sample hydration. Amplitude sweep experiments were performed in the range of 1–200% strain at a 5 Hz frequency to identify the linear viscoelastic region.

Following these measurements, the *G*′ and *G*″ of gels formed in triplicate were measured at a strain of 1% (within the linear viscoelastic region for all gels) at a 5 Hz frequency. Statistical significance was determined via a one-way ANOVA with a Benjamini–Kreuger–Yekutieli correction.

#### Time Sweep

To perform time sweep experiments, gels were formed directly on the rheometry plate, as described in the general Gel Formation procedure. The solution of 8-arm-PEG-thiol was pipetted onto the plate with the measurement gap at 1 mm. A time sweep was then initiated at 1% strain at a 1 Hz frequency. After 15–20 s, a solution of the cross-linker was then carefully added and vigorously mixed by pipetting. Measurements were taken every 5 s for 1 h measurement time, at 25 °C with a solvent trap used to maintain sample hydration.

### Cell Viability Studies

Hydrogels (100 μL) were formed in triplicate at pH 7.4 over 24 h in a sterile 96-well plate, as described above. THP-1 human monocytes (DSMZ) were then seeded on top of the gels at a density of 2.5 × 10^4^ cells/well and cultured in RPMI medium + 10% FBS + 1% penicillin/streptomycin as previously described by Grey et al.^24^ Cells were harvested after 24, 48, and 72 h in culture and were incubated with annexin V binding buffer in addition to the washing media (BD Biosciences no. 556547), washed three times in 1× annexin V binding buffer, and incubated with 5 μL/well propidium iodide prior to flow cytometry analysis. Absolute cell numbers were calculated using countbright beads (Thermo no. C36995) and half sampling of wells at each time point. Measurements were compared to controls on tissue culture plastic.

## Results and Discussion

### Kinetic Studies

The rate of thiol addition strongly influences the material properties of the hydrogels formed via Michael addition. If addition is too fast then mixing of the gel precursors can be inefficient, leading to heterogeneous gels containing defects caused by air bubbles and locals differences in thiol concentration.^15^ This heterogeneity has been previously identified as a challenge during thiol-maleimide cross-linking (rate of conjugation in model systems, *k*_1_ ∼ 10^3^ M^–1^ s^–1^).^16^ Conversely, if reaction rates are slow, then gelation rates suffer as a consequence, and the end materials often possess weak mechanical properties due to incomplete cross-linking (e.g., thiol-acrylamide gels, *k*_1_ ∼ 10^–2^ M^–1^ s^–1^).^17^ It was therefore important to characterize the reactivity of the PDs to be used in this work.

**Figure 2:**
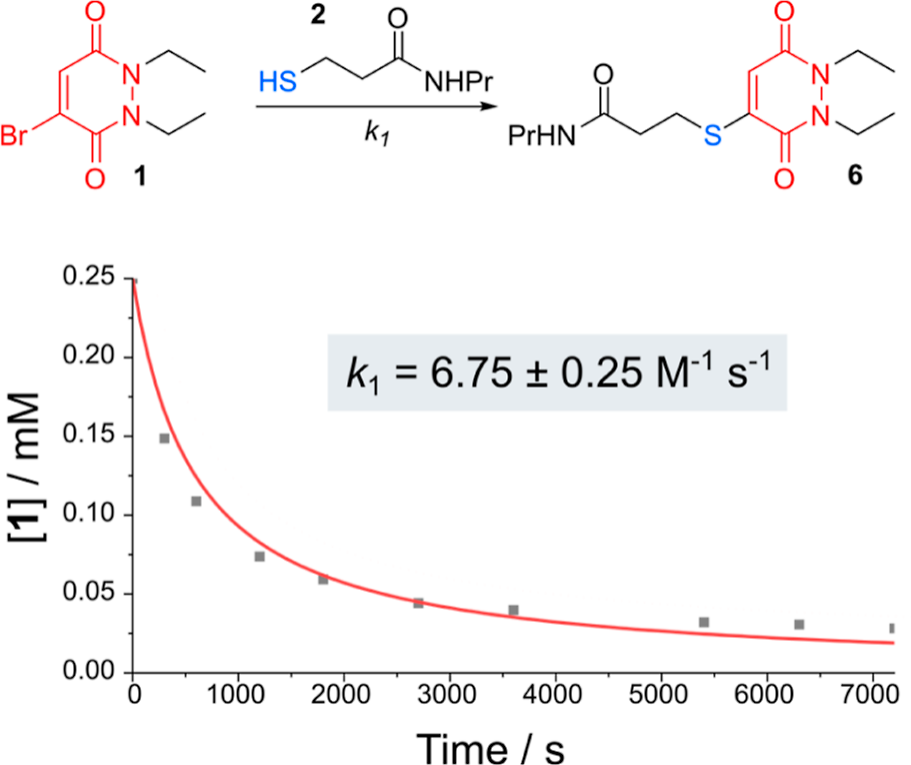
(a) Kinetic plot showing reaction of PD **1** with thiol **2** under second order conditions. *k*_1_ was determined from a plot of 1/[**1**] as described in the Supporting Information.

We have previously shown that monoBr-PDs have intermediate reactivity between faster reacting DiBr-PDs and slower reacting DiH-PDs.^13,14^ We therefore chose to study the rate of reaction of monoBr-PD **1** with thiol **2** as a model system (Figure 2). Reactions were undertaken under second-order conditions at a concentration of 0.25 mM at pH 7.4, and progress was monitored over time via liquid chromatography.

Absorption at 214 nm was used to monitor the concentration of **1** over time and to thus calculate *k*_1_ ∼ 6.75 M^–1^ s^–1^ (see Supporting Information, Figure S1). This makes the rate of mono-BrPD reactivity comparable to that of vinyl sulfones (*k*_1_ ∼ 10^–1^ M^–1^ s^–1^),^18^ which have been widely used for hydrogel cross-linking, and 2 orders of magnitude faster than commonly used acrylates and acrylamides.^19^ Given the relative reactivities of DiH-, monoBr-, and DiBr-PDs, we therefore expected that gelation would be possible with all three motifs, with the ability to tune gelation speed and hydrogel properties based on the cross-linker structure.

### Cross-Linker Synthesis

**Scheme 1:**
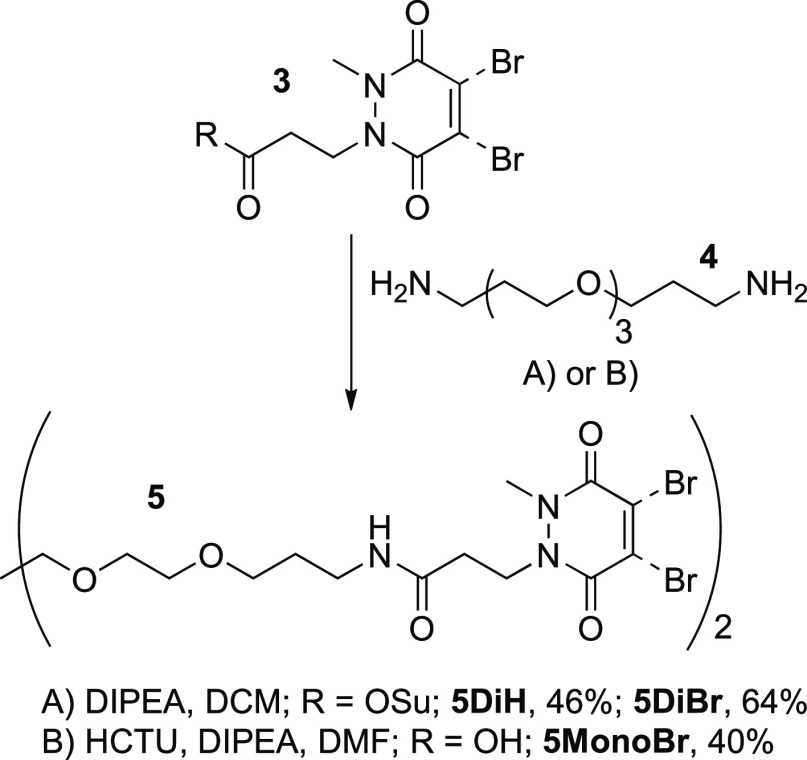
Synthesis of Bis-PD Crosslinkers **5**

We envisaged synthesizing bis-PDs as cross-linkers for the gelation of complementary thiol-capped star PEG macromers (Figure 1b). A short ethyleneglycol unit was integrated into the cross-linker design to provide flexibility and solubility, with amide couplings between carboxyl-, or *N*-hydroxysuccinimide ester-PDs **3** and 4,7,10-trioxa-1,13-tridecanediamine **4** delivering cross-linkers **5** in yields of 40–64% (Scheme 1). Of note, these cross-linkers could be obtained from commercially available starting materials in 3–4 steps using standard and straightforward synthetic techniques that would be accessible to researchers in most material science laboratories, without the need to exclude water or oxygen, or work with toxic, pyrophoric, or explosive chemicals (see the Supporting Information for full details).

All of the synthesized cross-linkers were soluble in water at low concentrations. However, at the concentrations needed for hydrogelation (50 mM), we found that solubilization was difficult. We therefore dissolved each cross-linker in a small amount of DMSO (0.4 M, leading to 5% w/v DMSO in final gels) prior to use. For **5DiH** and **5MonoBr**, solubility was maintained following dilution with buffer to the working concentrations needed for gel formation. However, dilution of DMSO stocks of **5DiBr** did lead to the formation of an opaque solution, though the homogeneity of this solution and the absence of precipitation allowed us to still carry this cross-linker forward for hydrogel formation. Stock solutions of the cross-linkers were found to be stable for >1 year with storage at −20 °C without needing any efforts to exclude oxygen or moisture.

### Hydrogelation

In an initial gelation test, **5MonoBr** was mixed with a 10 kDa 8-arm PEG-thiol, **6**, in pH 7.4 phosphate buffer and a final polymer content of 5% w/v. An equimolar ratio of thiol:PD was ensured to maximize cross-linking within the polymer network generated. Pleasingly, self-supporting gels were found to form after 2 h of incubation at room temperature. We therefore set out to study the gelation behavior of cross-linkers **5DiH**, **5MonoBr**, and **5DiBr** across a pH range of 6–8.

**Figure 3:**
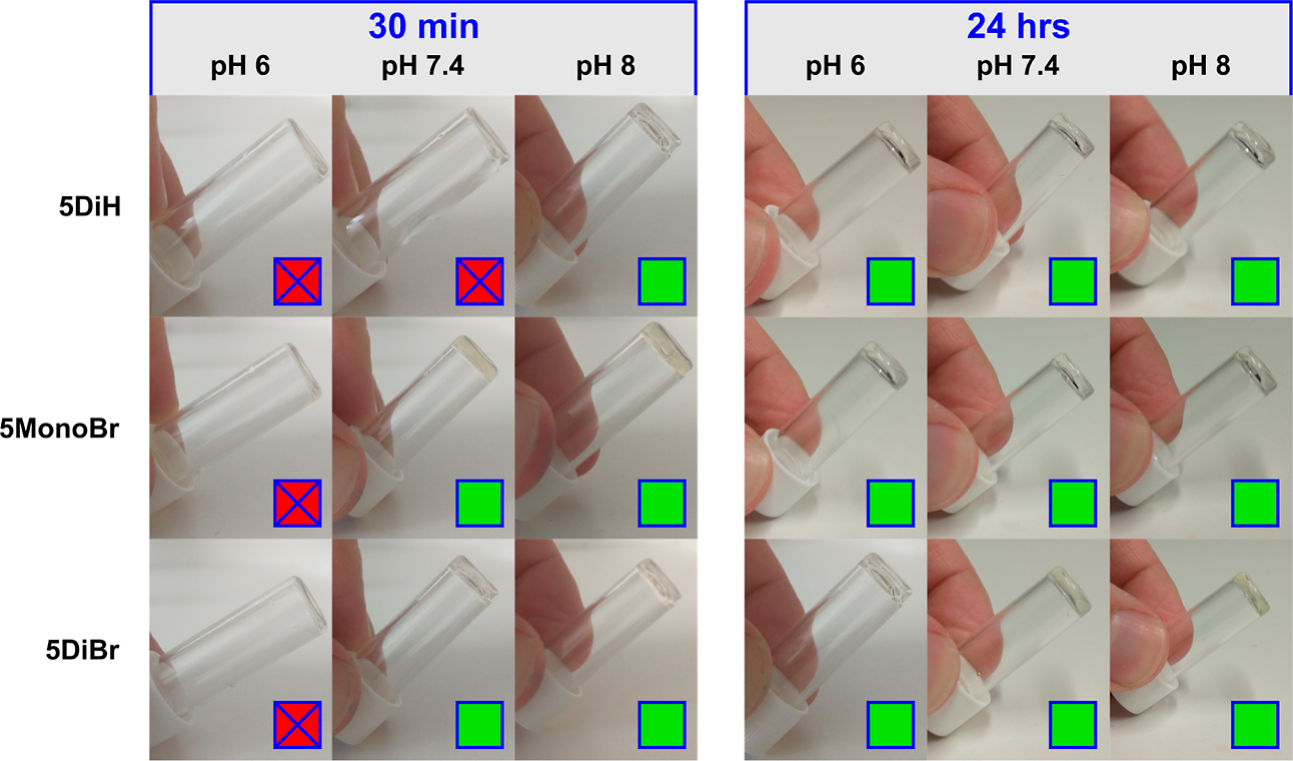
Inverted vials demonstrating either gel (green) or liquid (red) state following mixing of 8-arm PEG-SH, **6**, and PD cross-linkers **5** at different pHs and time points.

**5MonoBr** and **5DiBr** were both found to rapidly induce gelation at pH 7.4 and pH 8, with self-supporting materials formed after 30 min (Figure 3). At pH 8, **5DiH** was also able to form gels within 30 min, but at pH 7.4, the mixture remained liquid. This can be rationalized by the slower rate of reactivity of DiH-PDs relative to their brominated analogues. Analogously, no gels were formed after 30 min with any of the cross-linkers at pH 6, reflecting the low concentration of nucleophilic thiolates able to undergo Michael addition at this pH. In all cases, no change in the pH of the media was observed following gelation.

After 24 h, gels were formed under all conditions. This demonstrated that while the slower reactivity of **5DiH**, and of all cross-linkers under more acidic conditions, slowed gelation, sufficient cross-linking to form a robust, solution-spanning polymer network was still achievable. Control reactions in the absence of PD cross-linkers at pH 7.4 gave viscous solutions but no gel formation, while at pH 6, no significant increase in viscosity was observed. This result shows that background disulfide formation over long gelation periods is not a significant contributor to gel formation, although possible contributions supporting the PD-thiol network cannot be ruled out.

### Gel Characterization

**Figure 4:**
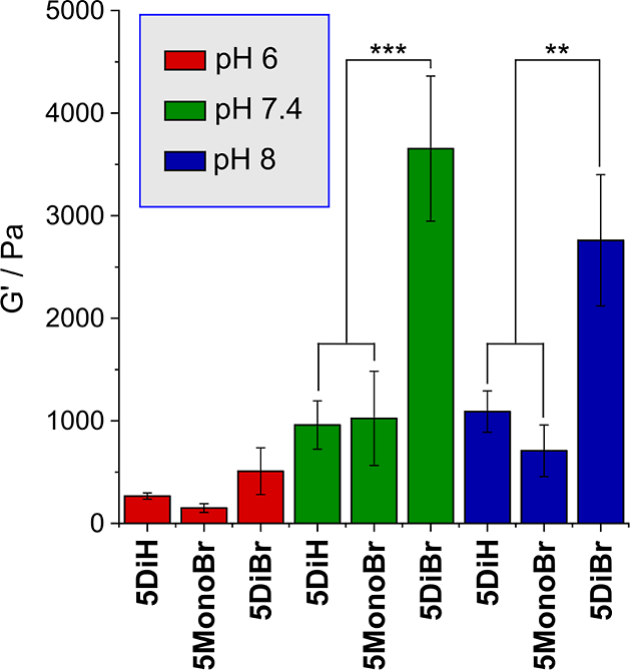
Plot of the storage modulus, *G*′, for gels formed from cross-linkers **5** at different pHs. One-way ANOVA with Benjamini–Kreuger–Yekutieli correction was used to calculate significance, **P ≤ 0.005, ***P ≤ 0.0005.

Having qualitatively observed gel formation, we next characterized the materials produced in more detail. All gels formed were sufficiently mechanically robust to be transferred to a parallel plate rheometer for further study. An amplitude sweep from 1 to 100% strain showed that gels formed under all conditions retained linear viscoelastic behavior up to a minimum of 10% strain (see Supporting Information, Figure S2). Storage and loss moduli of gels formed in triplicate were then calculated within this linear viscoelastic region (Figure 4). The results obtained reveal two interesting features:

i Gels formed at pH 6 were considerably weaker than those formed at pH 7.4 and 8. Differences in *G*′ at these two higher pHs were not significant. This result indicated that while a self-supporting network can be created at pH 6, the structure of this network is not fully cross-linked even after 24 h, leading to average values of *G*′ ranging from just 150–508 Pa.
ii Gels formed from **5DiBr** were stronger than those formed from **5DiH** and **5MonoBr** under all conditions. For example, while **5DiBr** generated gels with *G*′ = 3650 Pa at pH 7.4, those formed from **5DiH** and **5MonoBr** had *G*′ = 960 and 1024 Pa, respectively. A possible explanation for this increased mechanical strength could come from the ability of DiBr-PDs to undergo double-thiol addition^12^—although the thiol:PD stoichiometry was equimolar during gel formation, as network formation increases and reagent motility decreases, the formation of a perfect network where all thiols are consumed within productive cross-links becomes increasingly difficult.^20^ The presence of additional sites to consume these free thiols may contribute to the high mechanical strength observed for DiBr-PD cross-linked gels. We therefore measured the residual free thiol content in the gels, which was found to be ∼6% for gels formed from **5DiH**, and ∼1% for both **5MonoBr** and **5DiBr**. The higher levels of free thiol for **5DiH** can be rationalized by our previous observations that the reaction between DiH-PDs and thiols is dynamically reversible. Though the estimated rate of retro-Michael addition is relatively slow (∼10^–5^ s^–1^), within the confines of a gelated polymer network with restricted motion, this would be expected to lead to significant levels of free thiol at any one time.^13^ However, similarities in thiol content between **5MonoBr** and **5DiBr** gels suggest the capacity of DiBr-PDs to undergo dithiol addition is unlikely to be a major contributing factor to the enhanced mechanical strength.

To investigate the properties of the gels further, we undertook swelling studies on lyophilized gels formed from each of the three cross-linkers. The swelling ratio was found to be significantly lower when **5DiBr** was used as cross-linker (2000%) than corresponding gels formed from **5DiH** (4100%) or **5MonoBr** (3300%) (see Supporting Information, Figure S3). Though the **5DiBr** cross-linker itself is more hydrophobic than **5DiH** or **5MonoBr**, once integrated into the macromolecular architecture of the polymer network, it is unlikely this difference in properties is significant enough to lead to such large-scale differences in bulk scale behavior. Scanning electron microscopy (SEM) was also performed on the gels, with differences in the architecture of the lyophilized polymer network observable (see Supporting Information, Figure S5). Gels formed from **5MonoBr** were found to possess a web-like highly porous architecture, while those formed from **5DiH** and **5DiBr** were found to be more densely structured.

### Time Course of Gel Formation

**Figure 5:**
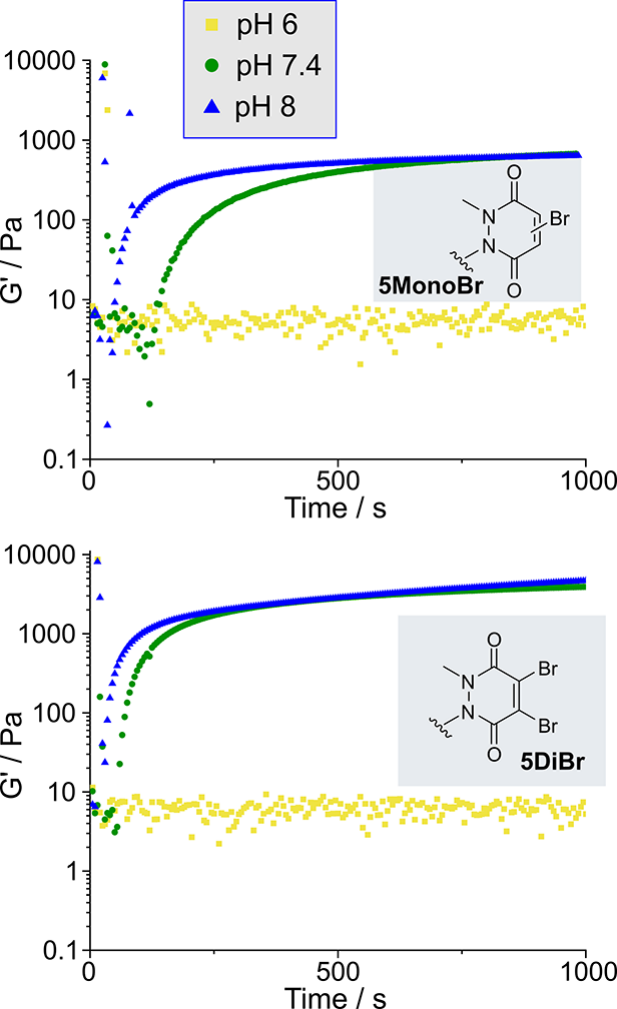
Plot of the storage modulus, *G*′, over time following mixing of 8-arm PEG-SH and PD cross-linkers **5**.

To study the gelation process further, we undertook time-sweep rheology measurements to monitor the evolution of *G*′ over time. Based on our observation that gels formed from **5DiH** were far slower to form, we focused on the use of **5MonoBr** and **5DiBr** as cross-linkers. The gel precursors were mixed directly on the rheometer plate and then *G*′ and *G″* measured over the course of 50 min. Measurements were performed at pH 6, 7.4, and 8, under conditions analogous to those in our initial gelation studies (Figure 5). As expected, gelation occurred rapidly at pH 8 for both cross-linkers (gelation point, defined as point at which *G*′ > *G″*, 25 s for **5MonoBr**, < 10 s for **5DiBr**). At pH 7.4, gelation was slightly slower with gelation points of 110 s for **5MonoBr** and 35 s for **5DiBr**. As such, **5DiBr** was found to induce slightly faster cross-linking than **5MonoBr** at both pHs, though differences were small. Furthermore, far higher final values of *G*′ were reached, as expected based on our previous results. At pH 6, no significant increase in *G*′ was observed over the course of the experiment, in accordance with our initial observations that gelation took ∼24 h at lower pH.

When comparing these results to gels previously formed from PEG-vinyl sulfones, quantitative differences cannot be deduced due to significant variation between experimental setups. However, qualitative observations suggest that gelation is more rapid in our brominated-PD systems, in line with the slightly increased reaction rates calculated for thiol-monoBrPD conjugation relative to previously reported values for vinyl sulfone addition (*k*_1_ ∼ 5–10 M^–1^ s^–1^ vs ∼1 M^–1^ s^–1^).^21^

### Thiol-Induced Gel Degradation

Our previous research has shown that the reaction of DiH-PDs with thiols is dynamically reversible, with the rate of retro-Michael addition estimated to be ∼10^–5^ s^–1^.^13^ Reversible, or dynamic, covalent cross-linking chemistries are of high value to the biomaterial community, providing opportunities to form stimuli-responsive hydrogels with applications in drug delivery, degradable scaffolds, and self-healing materials.^22,23^ We therefore considered whether our DiH-PD cross-linked hydrogels could be degraded by added thiols. At equilibrium, the balance of Michael and retro-Michael additions within the polymer network would be sufficient to maintain the gel structure, but upon addition of sufficient quantities of a small molecule thiol, the small amounts of unconjugated PD would start to be trapped outside of the network. Over time, this would lead to a breakdown of the polymer network and a loss of gel structure.

**Figure 6:**
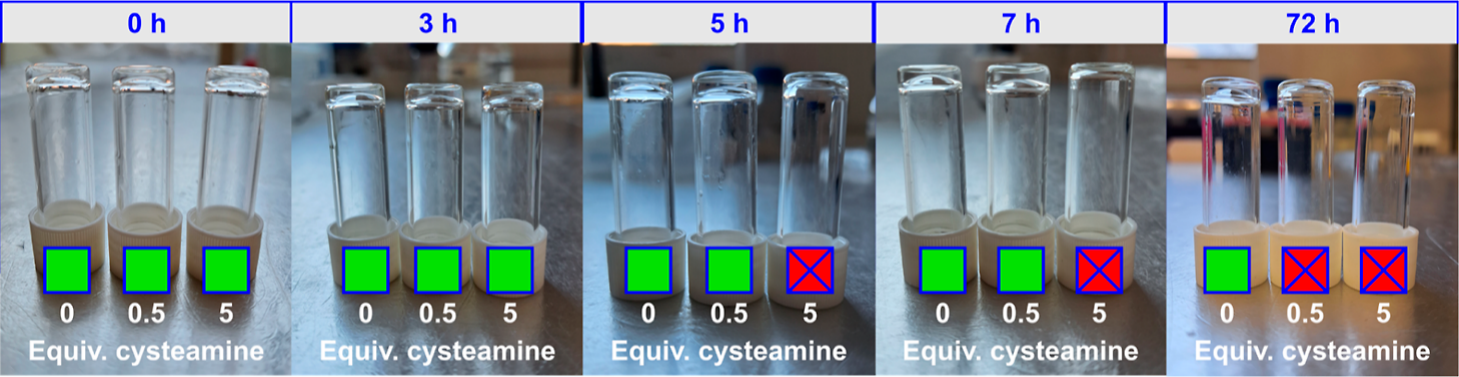
Inverted vials demonstrating either the gel (green) or liquid (red) state following incubation of gels formed from **5DiH** with different equivalents of cysteamine.

To investigate this, gels were preformed with **5DiH** and then solutions containing different quantities of cysteamine (0, 2, 20 μmol) were added on top. The gels were incubated at room temperature, and gel stability over time was monitored by inversion (Figure 6). After 5 h, it was found that the gel incubated with 20 μmol of cysteamine (∼5 equiv of thiol with respect to PD) had fully lost its structural integrity. In the presence of 2 μmol of cysteamine, degradation was far slower, with the gel only losing structural integrity after 96 h. Since this only equated to 0.5 equiv of thiol relative to PD, this supports the hypothesis that the polymer network within DiH-PD gels is incomplete, meaning only partial disruption is required to induce the loss of bulk structure.

### Cell Viability Studies

**Figure 7:**
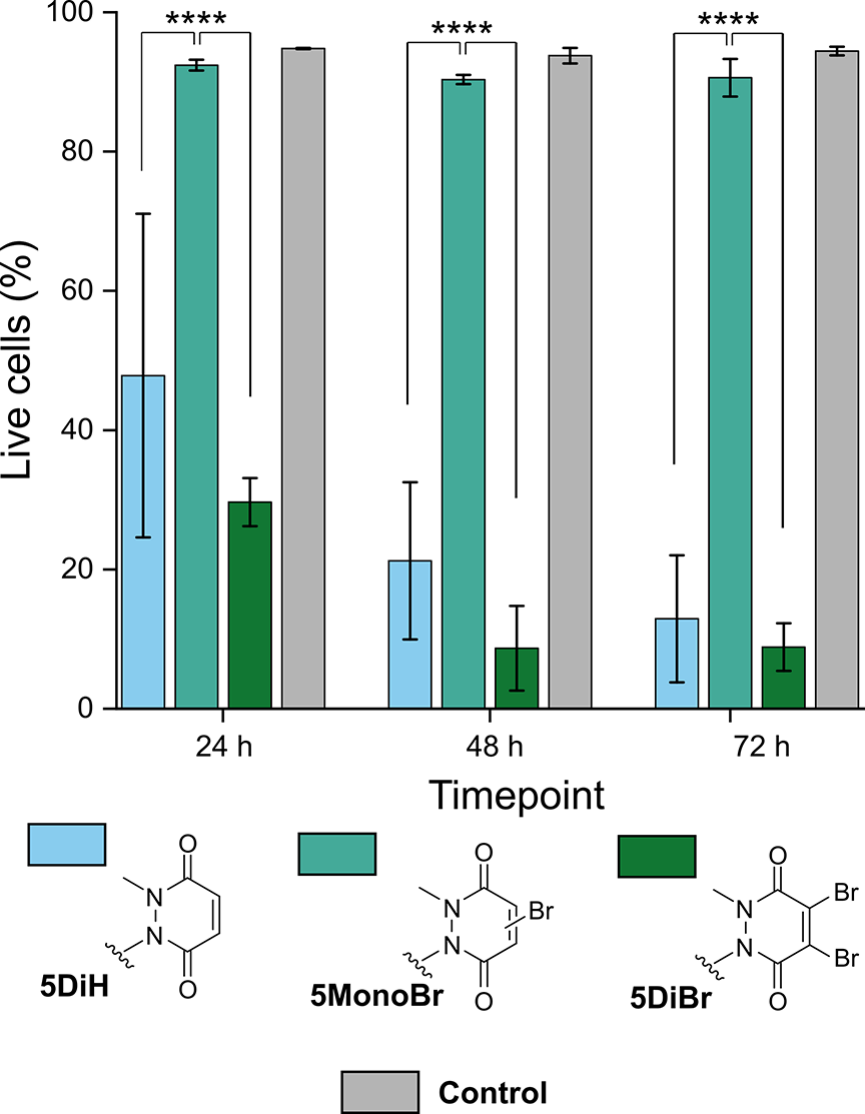
Plot of percentage live cells over time after seeding THP-1 cells on **5**-cross-linked hydrogels, or a tissue culture plastic control. Two-way ANOVA with Benjamini–Kreuger–Yekutieli correction was used to calculate the significance, ****P ≤ 0.00005.

Hydrogels are widely used in the biomedical community to scaffold cell growth and proliferation. To investigate the potential use of PD-cross-linked gels in this area, we therefore performed preliminary studies of cell viability. THP-1 human monocytes were seeded on to gels cross-linked with bisPDs-**5** at pH 7.4 and viability assessed after 24, 48, and 72 h.^24^ Interestingly, cell survival was found to depend on the cross-linker used. When gels were cross-linked with **5DiH** or **5DiBr**, a loss of viability was observed over 24 h. However, in contrast gels formed from **5MonoBr** were able to support high levels of survival and proliferation across the whole time period (91% survival over 72 h), to an equivalent level as a tissue culture plastic positive control (Figure 7 and Supporting Information, Figures S6 and S7). These promising results highlight potential future applications of PD-cross-linked gels as scaffolds for human cell culture.

## Conclusions

In this paper, we have introduced PDs as novel thiol-reactive cross-linking motifs for the formation of hydrogels. Importantly, these PDs address some of the drawbacks of the Michael acceptors that are currently widely used for hydrogel synthesis. In contrast to maleimides, PDs do not undergo hydrolysis at pH 6–8, while we have shown that reaction rates (and thus gelation rates) are orders-of-magnitude higher than for acrylates and acrylamides. Moreover, for mono- and DiBr-PDs our cross-linking chemistry is mechanistically resistant to retro-Michael addition, providing further benefits over the Michael acceptors that are most commonly employed in the biomaterials community. The tunable properties and rates of gelation offered by choice of DiH-, monoBr-, or DiBr-PD are also attractive, making PDs a valuable addition to the toolbox of reagents for hydrogel formation.

## Data Availability

Data associated with the study is available on the University of York Research Database, https://doi.org/10.15124/4143bae2-c299-4591-8644-dd39fad99eb2.

## Supporting Information Available

All details of reagent synthesis and procedures of all experiments not detailed in the Experimental Section (PDF)
