# Supplementary material for: Hydrogel Cross-Linking via Thiol-Reactive Pyridazinediones
Source: Biomacromolecules. 2023 Oct 4;24(11):4646–52. doi: 10.1021/acs.biomac.3c00290 (PMC10646975; doi:10.1021/acs.biomac.3c00290)
Supplement: Supplementary file 1 — bm3c00290_si_001.pdf [file bm3c00290_si_001.pdf]

Hydrogel crosslinking via thiol-reactive pyridazinediones

Calise Bahou,[†a] Richard J. Spears, [†a] Angela Ramírez Rosales,[b,c] Léa N. C. Rochet,[a] Lydia J. Barber,[b,c] Ksenia S. Stankevich,[b,c] Juliana F. Miranda,[c] Adam M. Kerrigan,[d] Tobias C. Butcher,[a] Vlado K. Lazarov,[e] William Grey,[c] Vijay Chudasama,[*a] and Christopher D. Spicer*[b,c]

[a]Department of Chemistry, University College London, 20 Gordon Street, London, WC1H 0AJ, UK
[b]Department of Chemistry, University of York, Heslington, YO10 5DD, UK
[c]York Biomedical Research Institute, University of York, Heslington, YO10 5DD, UK
[d]The York JEOL Nanocentre, University of York, Heslington, YO10 5BR, UK
[e]School of Physics, Engineering and Technology, University of York, Heslington, YO10 5DD, UK

Table of contents

S2 Supplementary figures

S5 General considerations

S6 Crosslinker synthesis

S11 Other synthesis

S12 Small molecule kinetic models

S13 Quantification of free thiols

S13 Swelling ratio calculation

S14 Scanning electron microscopy

S14 Gel degradation by competitive thiols

S14 Comparison of PD-gelation strategy to previously reported vinyl sulfones

S15 References

S16 NMR spectra of novel compounds

Supplementary figures

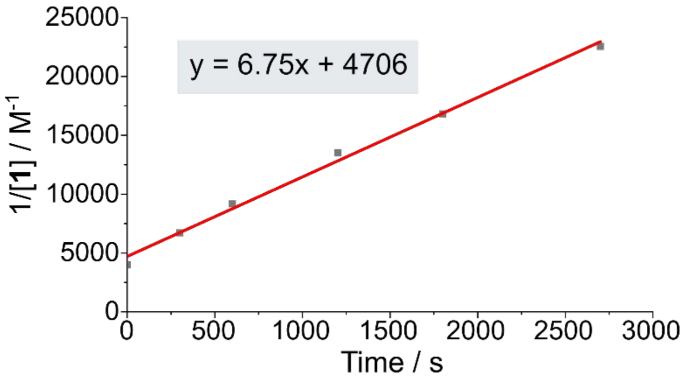

Figure S1: Plot of inverse [**1MonoBr**] against time following the reaction of **1MonoBr** with **2** under second order conditions at a concentration of 250 µM (grey squares), and linear fit of the data (red line, R^2 = 0.99).

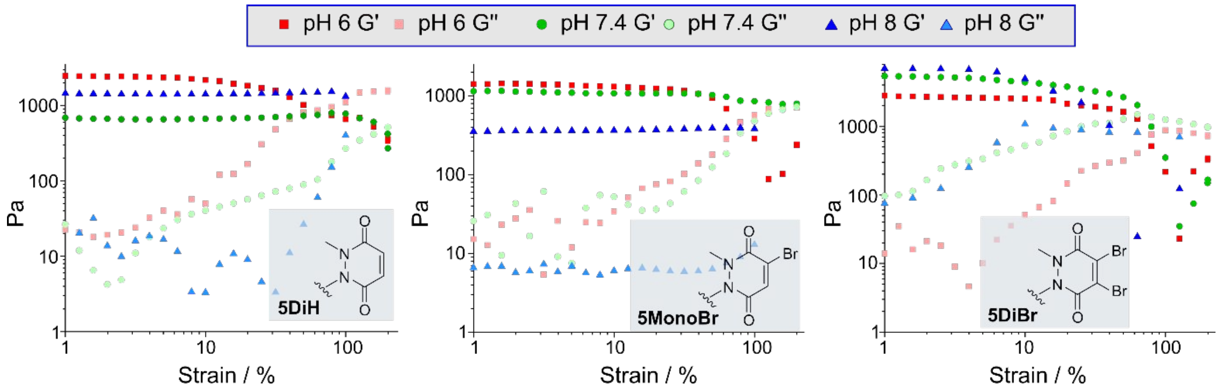

Figure S2: Amplitude sweep rheology measurements of gels crosslinked with bis-PDs **5** at a frequency of 5 Hz.

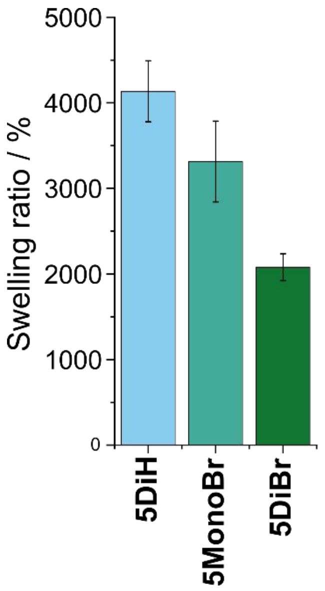

Figure S3: Plot of swelling ratios for gels crosslinked with bis-PDs **5** in PBS after 72 hrs.

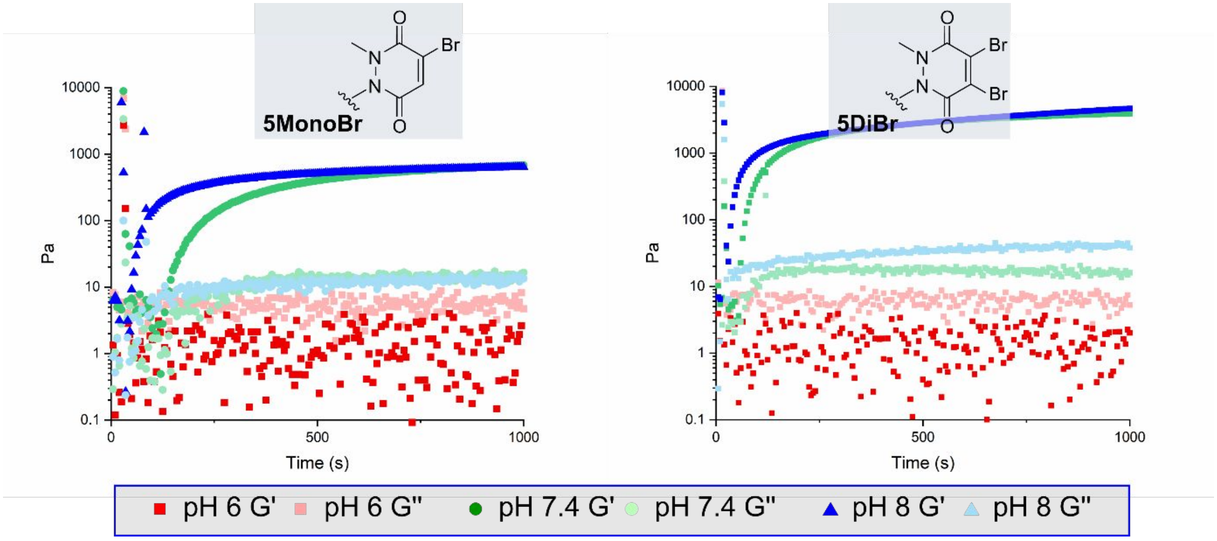

Figure S4: Time sweep rheology measurements at 1% strain and 1 Hz frequency, following mixing of 8-arm PEG-SH and PD crosslinkers **5**.

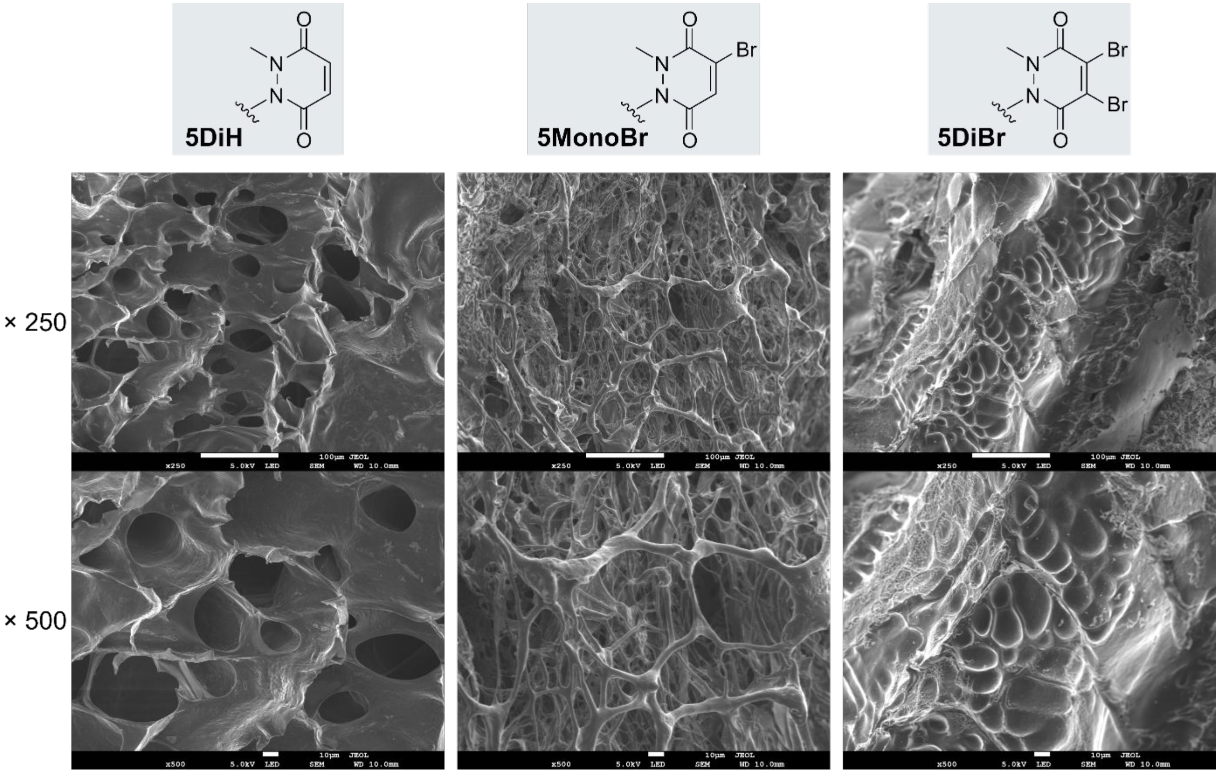

Figure S5: Representative SEM images of lyophilised gels formed at pH 7.4 with each crosslinker. Images are taken at ×250 magnification (scale bar: 100 µm) and ×500 magnification (scale bar: 10 µm).

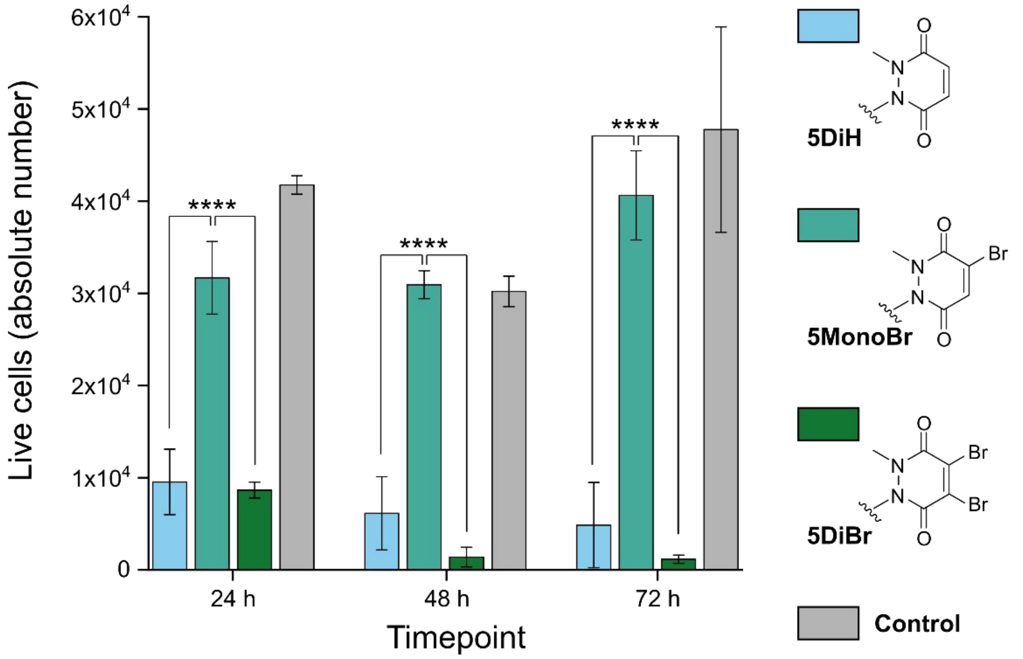

Figure S6: Plot of absolute cell numbers over time after seeding THP-1 cells on **5**-crosslinked hydrogels, or a tissue culture plastic control. test

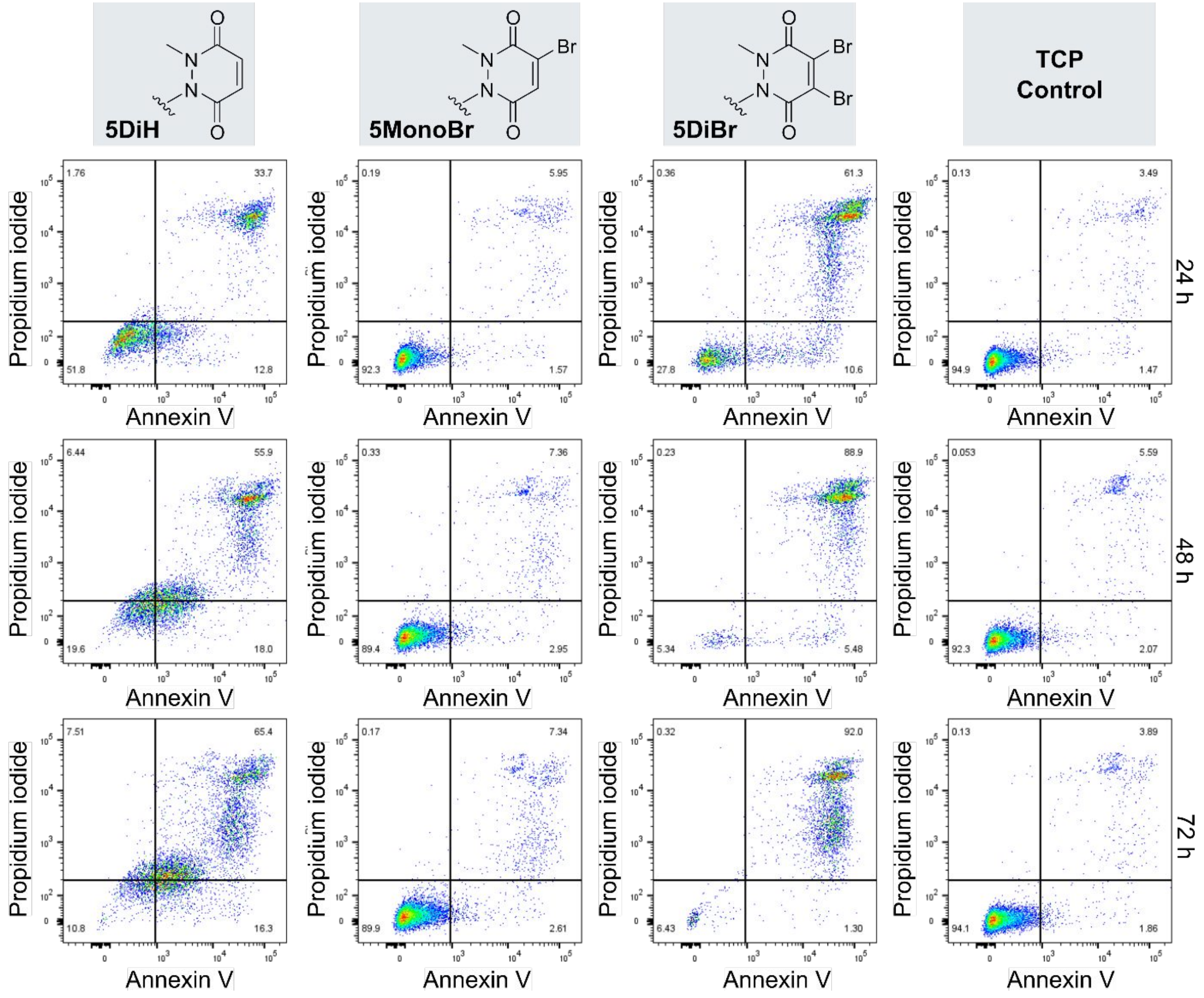

Figure S7: Representative flow cytometry data of three replicates for cell viability studies of THP-1 cells cultured on **5**-crosslinked hydrogels or tissue culture plastic controls.

General considerations

Proton and carbon nuclear magnetic resonance (^1H and ^{13}C NMR respectively) spectra were recorded on Bruker AvanceIII 400 (400 MHz), Avance III 600 (600 MHz), or Avance Neo 700 (700 MHz) spectrometers. NMR shifts were assigned using COSY, HSQC and HMBC spectra. All chemical shifts are quoted on the δ scale in ppm using residual solvent as the internal standard (^1H NMR: $CDCl_3$ = 7.26; DMSO-d_6 = 2.50 and ^{13}C NMR: $CDCl_3$ = 77.16, DMSO-d_6 = 39.52). Coupling constants (*J*) are reported in Hz with the following splitting abbreviations: s = singlet, d = doublet, t = triplet, q = quartet, m = multiplet, app = apparent, br = broad. Melting points (m.p.) were recorded on a Gallenkamp melting point apparatus and are uncorrected. Infrared (IR) spectra were recorded on a Perkin Elmer Spectrum 100 FTIR Spectrometer. Absorption maxima (υ_{max}) are reported in wavenumbers (cm^{-1}). High resolution electrospray ionisation (ESI) mass spectra (HRMS) were recorded on a Waters LCT Premier XE or a Bruker Compact TOF-MS spectrometer. Nominal and exact *m/z* values are reported in Daltons (Da). Rheological measurements were performed on a Malvern Instruments Kinexus Pro$^+$ Rheometer fitted with a 8 mm parallel plate geometry.

Thin layer chromatography (TLC) was carried out using aluminium backed sheets coated with 60 F_{254} silica gel (Merck). Visualization of the silica plates was achieved using a UV lamp (λ_{max} = 254 nm), potassium permanganate (5% $KMnO_4$ in 1M NaOH with 5% potassium carbonate), or ninhydrin (1.5% ninhydrin, 3% AcOH in *n*-butanol). Flash column chromatography was carried out with pre-loaded GraceResolv™ Silica Flash Cartridges (Grace™) or FlashPure EcoFlex cartridges (Büchi) on a Biotage Isolera Spektra One flash chromatography system (Biotage). Mobile phases are reported as % volume of more polar solvent in less polar solvent. Deionized water was used for chemical reactions and for protein manipulations. All other solvents were used as supplied (Analytical or HPLC grade), without prior purification. Reagents were purchased from Sigma-Aldrich, VWR, or Fluorochem and used as supplied, unless otherwise indicated. Brine refers to a saturated solution of sodium chloride. Petrol

refers to the fraction of petroleum ether boiling in the range 40-60 °C. Anhydrous magnesium sulfate ($MgSO_4$) was used as the drying agent after reaction workup unless otherwise stated.

1. Crosslinker synthesis

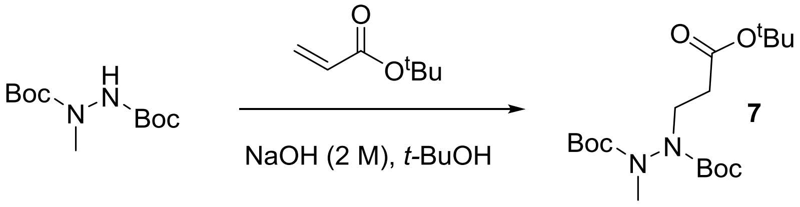

To a solution of di-*tert*-butyl-1-methylhydrazine-1,2-dicarboxylate (2.50 g, 10.1 mmol) in *tert*-butanol (20 mL), was added 2 M NaOH (0.34 mL) and the reaction mixture stirred at 21 °C for 10 min. After this, *tert*-butyl acrylate (4.41 mL, 30.4 mmol) was added and the reaction mixture was refluxed for 72 h. After cooling to room temperature, the solvent was removed *in vacuo* and the crude residue was dissolved in EtOAc (150 mL) and washed with water (3 × 50 mL). The organic layer was then dried over $MgSO_4$, filtered, and concentrated *in vacuo* to afford di-*tert*-butyl-1-(3-(*tert*-butoxy)-3-oxopropyl)-2-methylhydrazine-1,2-dicarboxylate **7** (2.92 g, 7.77 mmol, 77%) as a yellow oil. ^1H NMR (600 MHz, $CDCl_3$, rotamers) δ 3.80-3.52 (m, 2H), 3.03-2.97 (m, 3H), 2.51-2.47 (m, 2H), 1.46-1.40 (m, 27H). ^{13}C NMR (150 MHz, $CDCl_3$, rotamers) δ 169.2 (C), 153.6 (C), 152.5 (C), 79.2 (C), 42.7 (CH_3), 34.8 (CH_2), 32.3 (CH_2), 26.5 (CH_3). IR (thin film) 2969, 2927, 1715 cm^{-1}. LRMS (ESI) 375 (100, $[M+H]^+$), 319 (30, $[M-C_4H_9+2H]^+$ HRMS (ESI+) calcd for $C_{18}H_{35}N_2O_6$ $[M+H]^+$ 376.2524; observed 376.2516.

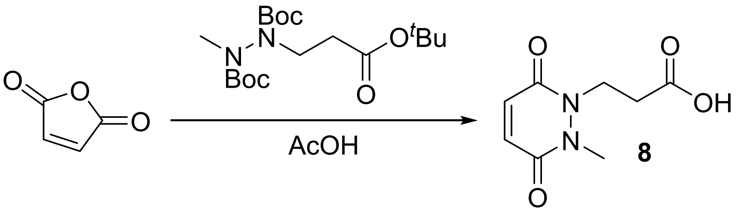

To a solution of maleic anhydride (157 mg, 1.61 mmol, 1.5 equiv.) in glacial AcOH (20 mL) was added di-*tert*-butyl 1-(3-(*tert*-butoxy)-3-oxopropyl)-2-methylhydrazine-1,2-dicarboxylate **7** (400 mg, 1.07 mmol, 1.0 equiv.). The reaction mixture was then refluxed for 18 h. After this time, the reaction mixture was allowed to cool to 21 °C, and the solvent was removed *in vacuo* with toluene co-evaporation (3 × 20 mL, as an azeotrope). Residual toluene was subsequently azeotroped with chloroform (3 × 20 mL). The residue was then purified via automated column chromatography (0-1%

MeOH/EtOAc, 1% AcOH) to give **7** as a cream solid (180 mg, 0.91 mmol, 85%). ^{1}H NMR (700 MHz, DMSO-d_6) δ 6.91 (q, *J* = 10.1 Hz, 2H), 4.22 (m, 2H), 3.50 (s, 3H), 2.57 (m, 2H); ^{13}C NMR (176 MHz, DMSO-d_6) δ 171.9 (C), 156.6 (C), 156.4 (C), 134.5 (CH), 134.2 (CH), 41.1 (CH_2), 32.4 (CH_3), 31.9 (CH_2); IR (thin film) 3078, 2741, 1716, 1607, 848 (cm^{-1}); HRMS (ES+) calcd for $C_8H_{11}N_2O_4^+$ [M+H]$^+$, 199.0719 observed 199.0715.

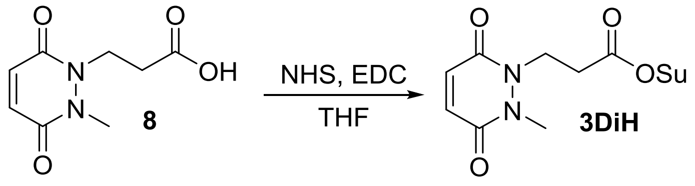

A solution of **8** (550 mg, 2.78 mmol, 1 equiv.) in THF (50 mL) was cooled to 4 °C under argon. *N*-(3-dimethylaminopropyl)-*N′*-ethylcarbodiimide hydrochloride (EDC. HCl, 1.067 g, 5.56 mmol, 2 equiv.) was then added and the mixture was stirred for 30 min. After this time, *N*-hydroxysuccinimide (639 mg, 5.56 mmol, 2 equiv.) was added and the reaction was allowed to stir at 21 °C for 16 h. The reaction mixture was then filtered and the filtrate was concentrated *in vacuo*. The crude residue was purified via automated column chromatography (50-100% EtOAc/Cyclohexane) to give **3DiH** as a light brown solid (635 mg, 2.16 mmol, 78%). ^{1}H NMR (600 MHz, $CDCl_3$) δ 6.93-6.86 (m, 2H), 4.43 (t, *J* = 7.1 Hz, 2H), 3.60 (s, 3H), 3.07-3.05 (m, 2H), 2.86-2.84 (m, 4H); ^{13}C NMR (150 MHz, $CDCl_3$) δ 168.8 (C), 166.1 (C), 157.5 (C), 157.4 (C), 135.3 (CH), 134.2 (CH), 41.2 (CH_2), 33.3 (CH_3), 29.2 (CH_2), 25.7 (CH_2); IR (thin film) 2936, 1817, 1736, 1625, 1583 (cm^{-1}).

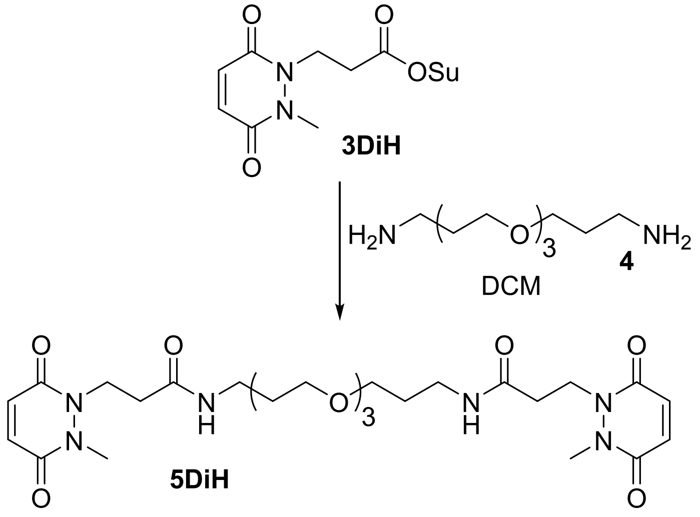

To a solution of **3DiH** (200 mg, 0.67 mmol, 4 equiv.) in DCM (30 mL) was added 4,7,10-trioxa-1,13-tridecanediamine **4** (37 µL, 0.17 mmol, 1.0 equiv.). The reaction mixture

was then stirred at 21 °C for 18 h. After this time, solvent was removed *in vacuo*, and the crude residue was purified via automated column chromatography (0-15% MeOH/DCM) to give **5DiH** as a yellow oil (45 mg, 0.08 mmol, 46%). ^{1}H NMR (600 MHz, $CDCl_3$) δ 6.90-6.83 (m, 4H), 4.40-4.37 (m, 4H), 3.66-3.65 (m, 6H), 3.62-3.60 (m, 4H), 3.56-3.52 (m, 8H) 3.35-3.32 (m, 4H), 2.58 (t, *J* = 7.1 Hz, 4H), 1.77-1.72 (m, 4H); ^{13}C NMR (150 MHz, $CDCl_3$) δ 169.5 (C), 157.2 (C), 157.2 (C), 135.0 (CH), 134.2 (CH), 70.5 (CH_2), 70.1 (CH_2), 70.0 (CH_2), 42.7 (CH_2), 38.2 (CH_2), 34.3 (CH_2), 33.1 (CH_3), 28.8 (CH_2); IR (thin film) IR (thin film) 2925, 2854, 1780, 1702, 1625, 1210, 1075, 652 cm^{-1}; HRMS (ES+) calcd for $C_{26}H_{40}N_6NaO_9$ $[M+Na]^+$ 603.2749, observed 603.2759.

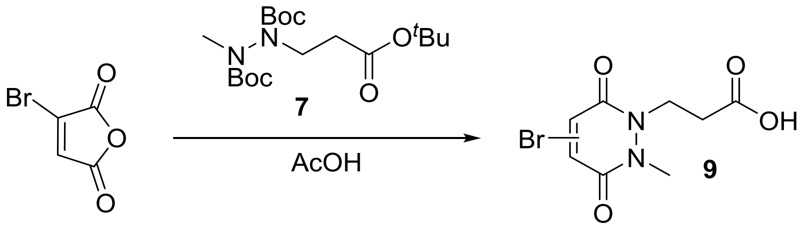

To a solution of di-*tert*-butyl 1-(3-(*tert*-butoxy)-3-oxopropyl)-2-methylhydrazine-1,2-dicarboxylate **7** (0.75 g, 2.01 mmol) in AcOH (10 mL) was added bromomaleic anhydride (0.21 mL, 2.21 mmol) and the reaction refluxed for 4 h. After cooling to room temperature, the reaction mixture was concentrated *in vacuo* with toluene co-evaporation (3 × 30 mL, as an azeotrope). The crude residue was then purified by flash column chromatography (0-10% MeOH:EtOAc, 1% AcOH) to afford an inseparable mixture of regioisomers of **9** (402 mg, 1.45 mmol, 72%) as a white solid.

^{1}H NMR (600 MHz, DMSO-d_6, regioisomers (1:1)) δ 7.59 (s, 1H), 7.58 (s, 1H), 4.28 (t, *J* = 7.4 Hz, 2H), 4.21 (t, *J* = 7.4 Hz, 2H), 3.58 (s, 3H), 3.50 (s,3H), 2.62–2.57 (m, 4H). ^{13}C NMR (150 MHz, DMSO-d_6, regioisomers (1:1)) δ 172.0 (C), 171.9 (C), 155.4 (C), 155.1 (C), 153.5 (C), 153.2 (C), 135.8 (CH), 135.5 (CH), 132.8 (C), 132.3 (C) 42.7 (CH_2), 41.5 (CH_2), 34.2 (CH_3), 32.9 (CH_3), 31.8 (CH_2). IR (solid) 3058, 1722, 1619 cm^{-1}.

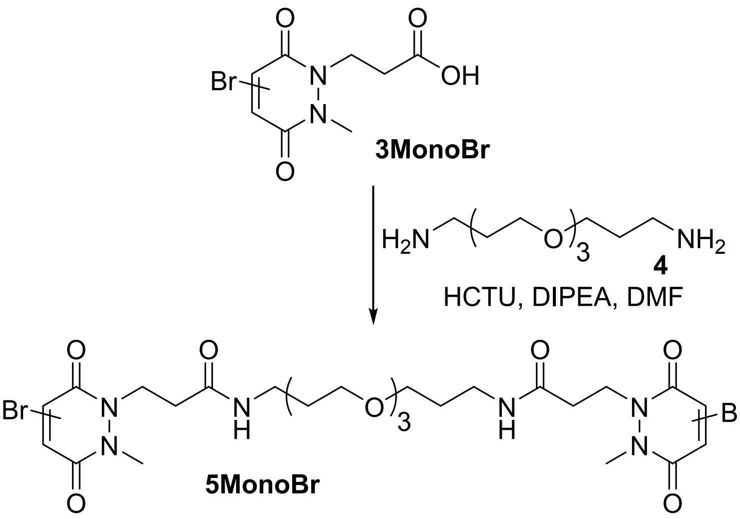

To a solution of mono-Br-PD acid **3MonoBr**[1] (100 mg, 0.44 mmol, 2.5 equiv.) and O-(1H-6-chlorobenzotriazole-1-yl)-1,1,3,3-tetramethyluronium hexafluorophosphate (HCTU, 182 mg, 0.44 mmol, 2.5 equiv.) in DMF (2 mL) was added 4,7,10-trioxa-1,13-tridecanediamine **4** (39 µL, 0.17 mmol, 1.0 equiv.) and *N*,*N*-diisopropylethylamine (77 µL, 0.44 mmol, 2.5 equiv.). The reaction mixture was then stirred at room temperature for 18 h. After this time, solvent was removed *in vacuo*, and the crude residue was purified via reverse-phase flash chromatography (5-100% MeCN:H_2O). Pure fractions were combined and concentrated *in vacuo* to give a mixture of regioisomers of mBr BisPD **5MonoBr** as an orange oil (53 mg, 0.07 mmol, 64%).

^{1}H NMR (700 MHz, $CDCl_3$): δ = 7.37 (s, 1H), 7.34-7.33 (m, 1H), 6.91-6.89 (m, 2H), 4.45-4.42 (m, 2H), 4.38-4.35 (m, 2H), 3.72-3.71 (m, 3H), 3.65-3.64 (m, 3H), 3.63-3.60 (m, 4H), 3.57-3.55 (m, 4H), 3.54-3.52 (m, 4H), 3.36-3.32 (m, 4H), 2.63-2.55 (m, 4H), 1.77-1.71 (m, 4H); ^{13}C NMR (176 MHz, $CDCl_3$) δ 169.3 (C), 155.9 (C), 154.0 (C), 139.2 (CH), 133.08 (C), 70.5 (CH_2), 70.0 (CH_2), 44.2 (CH_2), 43.07 (CH_2), 38.3 (CH_2), 34.4 (CH_3), 34.2 (CH_2), 33.5 (CH_2), 28.8 (CH_2); IR (thin film) 3304, 3002, 2866, 1625, 746 cm^{-1}; LRMS (ES+) 739 (100, $[M+H]^+$); HRMS (ES+) calcd for $C_{26}H_{39}Br_2N_6O_9^+$ $[M+H]^+$, 737.1145 observed 737.1135.

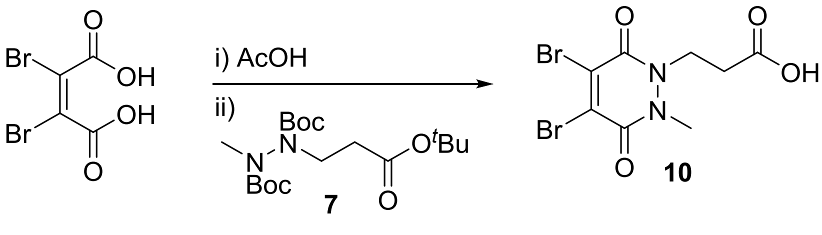

Dibromomaleic acid (0.88 g, 3.23 mmol) was dissolved in AcOH (37 mL) and refluxed for 30 min. To this solution, was added di-*tert*-butyl-1-(3-(tert-butoxy)-3-oxopropyl)-2-methylhydrazine-1,2-dicarboxylate **7** (1.45 g, 3.89 mmol) and the reaction was refluxed for a further 16 h. After cooling to room temperature, the reaction mixture was concentrated *in vacuo* with toluene co-evaporation (3 × 30 mL, as an azeotrope). The crude residue was purified by flash column chromatography, eluting with 20-100% EtOAc:cyclohexane, 1% AcOH, to afford **10** (0.80 g, 2.25 mmol, 70%) as a yellow solid. ^{1}H NMR (700 MHz, MeOD) δ 4.43 (t, *J* = 7.3 Hz, 2H), 3.68 (s, 3H), 2.73 (t, *J* = 7.3 Hz, 2H). ^{13}C NMR (150 MHz, MeOD) δ 173.8 (C), 154.7 (C), 154.5 (C), 136.7 (C), 136.4 (C), 44.9 (CH_3), 35.4 (CH_2), 32.5 (CH_2). IR (solid) 3044, 1729, 1603, 1572 cm-1

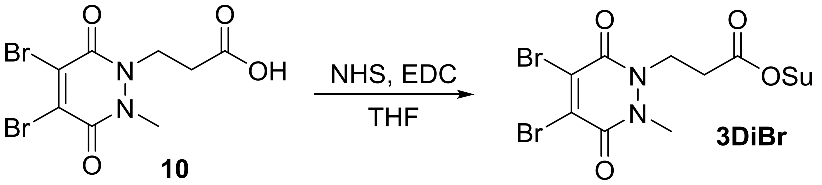

10

To a solution of **10** (0.70 g, 1.97 mmol) in dry THF (10 mL), was added 1-ethyl-3-(3-dimethylaminopropyl)carbodiimide.HCl (420 mg, 2.28 mmol) at 0 °C. After stirring for 30 min, *N*-hydroxysuccinimide (0.28 g, 2.95 mmol) was added and the mixture allowed to warm to 21 °C and stirred for a further 16 h. The reaction mixture was then concentrated *in vacuo* and the crude residue purified by flash column chromatography eluting with 20-100% EtOAc:cyclohexane. Pure fractions were concentrated *in vacuo* to afford **3DiBr** (0.35 g, 0.75 mmol, 38%) as a yellow solid. ^{1}H NMR (600 MHz, $CDCl_3$) δ 4.48 (t, *J* = 6.9 Hz, 2H), 3.67 (s, 3H), 3.10 (t, *J* = 6.9 Hz, 2H), 2.84 (s, 4H). ^{13}C NMR (150 MHz, $CDCl_3$) δ 169.2 (C), 168.8 (C), 166.1 (C), 153.4 (C), 153.1 (C), 136.9 (C), 135.4 (C), 43.0 (CH_2), 35.3 (CH_3), 29.1 (CH_2), 25.7 (CH_2); IR (solid) 3032, 2918, 1732, 1623, 1576, 643 cm^{-1}.

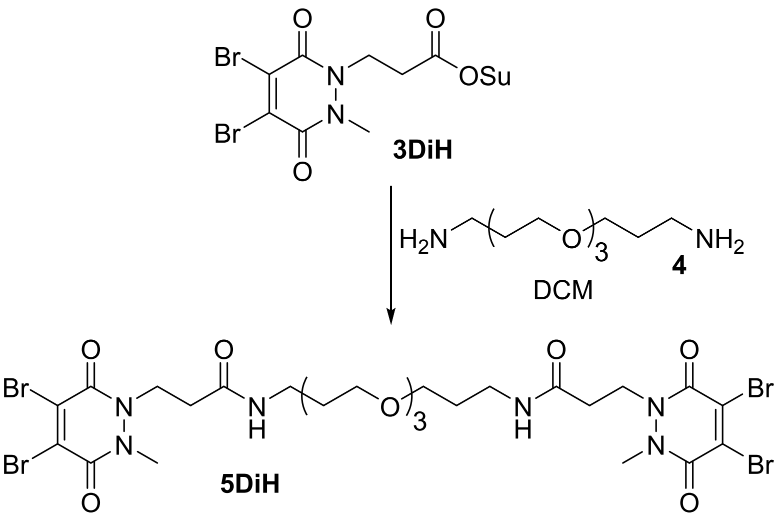

To a solution of **3DiBr** (100 mg, 0.221 mmol, 4.0 equiv.) in DCM (20 mL) was added 4,7,10-trioxa-1,13-tridecanediamine **4** (12 µL, 0.055 mmol, 1.0 equiv.). The reaction mixture was then stirred at 21 °C for 18 h. After this time, solvent was removed *in vacuo*, and the crude residue was purified via automated column chromatography (0-15% MeOH/EtOAc) to give **5DiBr** as a yellow oil (20 mg, 0.022 mmol, 40%). ^{1}H NMR (600 MHz, $CDCl_3$) δ 4.44 (t, *J* = 6.9 Hz, 4H), 3.73 (s, 6H), 3.62-3.60 (m, 4H), 3.56 – 3.52 (m, 8H), 3.35 – 3.32 (dd, *J* = 12.1, 5.8 Hz, 4H), 2.58 (t, *J* = 6.9 Hz, 4H), 1.74-1.70 (m, 4H); ^{13}C NMR (150 MHz, $CDCl_3$) δ 169.2 (C), 153.1 (C), 152.9 (C), 136.5 (C), 135.4 (C), 70.4, (CH_2) 70.2 (CH_2), 70.0 (CH_2), 44.5 (CH_2), 38.4 (CH_2), 35.3 (CH_2), 34.2 (CH_2), 28.7 (CH_3); IR (thin film) 3616, 2361, 1640, 1288, 683 cm^{-1}; LRMS (ES+) 897 (100, $[M^{81}Br^{81}Br^{79}Br^{79}Br+H]^+$); HRMS (ES+) calcd for $C_{26}H_{37}Br_4N_6O_9$ $[M^{79}Br^{79}Br^{79}Br^{79}Br+H]^+$ 892.9338, observed 892.9350

2. Other synthesis

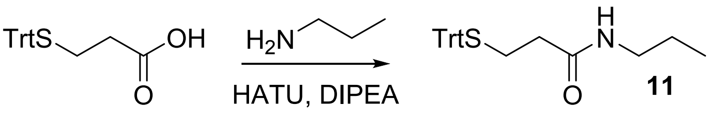

To a solution of 3-(tritylthio)propanoic acid (390 mg, 1.11 mmol, 1 equiv.) in DCM (8 mL) was added *O*-(7-azabenzotriazol-1-yl)-1,1,3,3-tetramethyluroniumhexafluorophosphate (HATU, 452 mg, 1.24 mmol, 1.1 equiv.), *n*-propylamine (102 µL, 1.24 mmol, 1 equiv.) and *N*,*N*-diisopropylethylamine (386 µL, 2.22 mmol, 2 equiv.). The reaction mixture was then stirred at room temperature for 20

h. After this time, the reaction was concentrated *in vacuo* and the crude residue was purified via flash column chromatography, eluting with 10-90% EtOAc:cyclohexane. Pure fractions were concentrated *in vacuo* to give *N*-propyl-3-(tritylthio)propenamide as a white solid (294 mg, 0.29 mmol, 68%). ^{1}H NMR (400 MHz, $CDCl_3$) δ 7.43 (m, 6H), 7.28 (m, 6H), 7.21 (m, 3H), 3.14 (dd, *J* = 13.4, 6.7 Hz, 2H), 2.50 (t, *J* = 7.3 Hz, 2H), 2.01 (t, *J* = 7.3 Hz, 2H), 1.46 (m, 2H), 0.88 (t, *J* = 7.4 Hz, 3H); ^{13}C NMR (125 Hz) δ 170.9 (C), 144.7 (C), 129.7 (CH), 128.0 (CH), 126.8 (CH), 66.9 (C), 41.3 (CH_2), 35.9 (CH_2), 27.9 (CH_2), 22.9 (CH_2), 11.4 (CH_3); IR (thin film) 3250, 3082, 1641, 1555, 698 (cm^{-1}); LRMS (ES+) 412 (100, $[M+Na]^+$); HRMS (ES+) calcd for $C_{25}H_{27}NNaOS^+$ $[M+Na]^+$, 412.1711 observed 412.1709

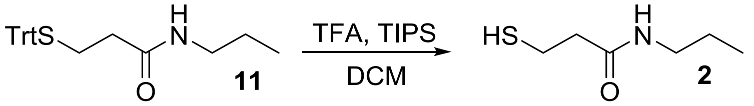

N-propyl-3-(tritylthio)propenamide **11** (140 mg, 0.36 mmol) was dissolved in a mixture of DCM:TFA:TIS (4 mL, 1:1:0.1) and the resulting solution was stirred at room temperature for 2 h. After this time, the reaction mixture was concentrated *in vacuo* and the crude residue was purified via flash column chromatography, eluting with 10-90% EtOAc:cyclohexane. Pure fractions were concentrated *in vacuo* to give 3-mercapto-*N*-propylpropanamide as a colourless oil (37 mg, 0.25 mmol, 70%). ^{1}H NMR (400 MHz, $CDCl_3$) δ 3.23 (dd, *J* = 13.4, 6.7 Hz, 2H), 2.81 (dd, *J* = 14.8, 6.9 Hz, 1H), 2.48 (t, *J* = 6.7 Hz, 1H), 1.61 (m, 1H), 1.55 (m, 2H), 0.93 (t, *J* = 7.4 Hz, 3H); ^{13}C NMR (125 MHz, CD_3Cl) δ 170.8 (C), 41.4 (CH_2), 40.6 (CH_2), 22.9 (CH_2), 20.6 (CH_2), 11.4 (CH_3); IR (thin film) 3294, 2964, 1640, 1548, 753 (cm^{-1}); LRMS (ES+) 148 (100, $[M+H]^+$); (ES+) calcd for $C_6H_{14}NOS^+$ $[M+H]^+$, 148.0796 observed 148.0798.

3. Small molecule kinetic models

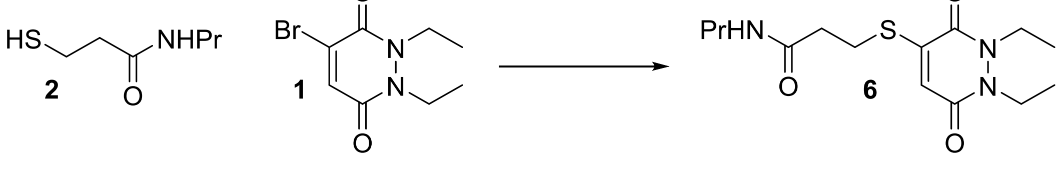

Stock solutions of 3-mercapto-*N*-propylpropanamide **2** (10 mM) and monoBrPD **1**[2] (10 mM) were prepared in acetonitrile. An aliquot of each stock (5 µL, xxx µmol) was added

sequentially to PBS (190 µL, pH 7.4 containing 2 mM EDTA) and the reaction mixture was incubated at 37 °C. At specified time points, a 5 µL aliquot of the reaction was removed and quenched with water (xxx µL, containing 1% formic acid) and analysed by UPLC-MS. Quantification of conversion to the product was assessed by integration of the peaks corresponding to the monoBrPD starting material and the product at A_{214} nm. The second order rate constant (k_2) was calculated from a plot of 1/[monoBrPD] vs time (see supplementary figure Fig. S1) and a linear fitting, giving a value of k_1 = 6.75 M^{-1} s^{-1}.

4. Quantification of free thiols

100 µL gels were formed over 24 hrs at pH 7.4, as described above. A stock solution of Ellman's reagent (100 µL, 2 mM in pH 7.4 phosphate buffer) was then added on top, and the gels incubated at room temperature for 20 min. The supernatant was then removed and absorbance measured at 405 nm. The quantities of free thiols in the gel were then calculated based on a comparison to a standard curve of solutions of 3-mercaptopropionic acid (1.6, 8, 40, 200, and 1000 µM) treated in an identical manner. Free thiol levels when different crosslinkers were used were determined to be: **5DiH** – 254 nmol, 6%; **5MonoBr** – 36 nmol, 1%; **5DiBr** – 46 nmol, 1.2%.

5. Swelling ratio calculations

Gels were formed over 24 hrs at pH 7.4, as described above, in pre-weighed 0.5 mL microcentrifuge tubes. After this time, water was added (400 µL) and the gels incubated for 2 hrs. The supernatant was then discarded and this process repeated 5 times to equilibrate the gels in pure water. The gels were then lyophilised and the dry mass measured. The gels were then swollen for 48 hrs in water (400 µL) and the swollen mass of the gels measured. The swelling ratio was calculated by dividing swollen and dry masses. Experiments were run in triplicates

6. Scanning electron microscopy

100 µL gels were formed over 24 hrs at pH 7.4, as described above. The gels were then washed with deionised water (3 × 1 h, 400 µL) and lyophilised. The samples were prepared for SEM imaging via cutting with a scalpel and affixing to an Al SEM stub via a carbon pad.

The SEM images were acquired on a JEOL JSM-7800F prime, equipped with a Schottky (field-assisted) thermionic emitter, at the York JEOL Nanocentre. An off-axis Everhart-Thornley detector with a positive bias, in LED mode 3, for attraction of both secondary and backscattered electrons was used. An objective lens aperture size of 30 µm was used with an accelerating voltage of 5 keV, resulting in a probe current of 0.1 nA. At the used working distance of 10 mm, with the aforementioned settings, the maximum resolution of the instrument is 3 nm. The images produced are 1280 x 960 pixels in resolution and were collected with a dwell time of 14 µs at each pixel.

7. Gel degradation by competitive thiols

After being left to form for 24 hrs, solutions of cysteamine at differing concentrations (200 µL; 0, 10, or 100 mM; 0, 0.5, or 5 equiv. thiol w.r.t. 8-arm-PEG-thiol precursor) were added to the top of DiH-PD crosslinked gels. The gels were incubated for 24 hrs, and at set times were inverted to assess gel stability and photographed.

8. Comparison of PD-gelation strategy to previously reported vinyl sulfones

In the main manuscript we highlight that quantitative comparisons to previously reported vinyl sulfone gels are difficult due to differences in experimental set-up. Specifically, in the prior work of Lutolf and Hubbell the gelation components are reversed with a vinyl-sulfone functionalised PEG macromer, and thiol-based crosslinker. This crosslinker is peptide-based, with N-terminal cysteine residues which are known to undergo differing reactivity to simple thiols. Moreover, gelation was performed in 0.3 M triethanolamine solutions at a desired pH, rather than phosphate buffer used in our work, which may affect gelation times and gel properties. However,

we suggest that qualitatively brominated-PDs appear to undergo gelation faster, in line with the higher rates of thiol conjugation (~1-10 M^{-1} s^{-1} vs. ~0.1 M^{-1} s^{-1} for vinylsulfones).

9. References

(1) Nogueira, J. C. F.; Greene, M. K.; Richards, D. A.; Furby, A. O.; Steven, J.; Porter, A.; Barelle, C.; Scott, C. J.; Chudasama, V. *Chem. Commun.* **2019**, *55*, 7671.

(2) Chudasama, V.; Smith, M. E. B.; Schumacher, F. F.; Papaioannou, D.; Waksman, G.; Baker, J. R.; Caddick, S. *Chem. Commun.* **2011**, *47* (31), 8781.

(3) Grey, W.; Rio-Machin, A.; Casado, P.; Grönroos, E.; Ali, S.; Miettinen, J. J.; Bewicke-Copley, F.; Parsons, A.; Heckman, C. A.; Swanton, C.; Cutillas, P. R.; Gribben, J.; Fitzgibbon, J.; Bonnet, D. *Sci. Transl. Med.* **2022**, *14* (650), eabn3248.
